# Contemporary Treatment of Silent Sinus Syndrome: A Case Report and Literature Review

**DOI:** 10.7759/cureus.57577

**Published:** 2024-04-04

**Authors:** Manuel Tousidonis, Sara Alvarez-Mokthari, Saad Khayat, Guillermo Sanjuan de Moreta, Santiago Ochandiano

**Affiliations:** 1 Oral and Maxillofacial Surgery, Gregorio Marañon University Hospital, Madrid, ESP; 2 Otolaryngology, Gregorio Marañon University Hospital, Madrid, ESP

**Keywords:** patient-specific implant, functional endoscopic sinus surgery, chronic maxillary atelectasis, enophtalmos, orbital volume, silent sinus syndrome

## Abstract

Silent sinus syndrome is a rare clinical entity affecting the maxillary sinus, characterized by ipsilateral enophthalmos and hypoglobus. Its etiology and pathophysiology are still debated. It is diagnosed by clinical examination and confirmed with computed tomography. It is commonly managed surgically. We present the case of a 34-year-old woman with silent sinus syndrome treated with a patient-specific implant for orbital reconstruction, functional endoscopic sinus surgery approach, intraoperative scan, and surgical navigation, successfully restoring orbital volume and sinus ventilation.

## Introduction

Silent sinus syndrome (SSS), first described by Montgomery in 1964 [1], is characterized by the spontaneous collapse of the maxillary sinus walls, resulting in the descent of the orbital floor. Clinical presentation typically includes painless progressive enophthalmos, hypoglobus, diplopia, orbital asymmetry, diminished malar projection, and facial asymmetry [2-6]. The precise etiology of this syndrome remains unknown, and the primary objective of treatment is to reinstate proper sinus ventilation and orbital volume [3-7].

## Case presentation

**Figure 1:**
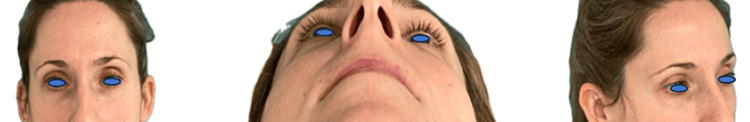
Preoperative images showing left eye hypoglobus and enophthalmos.

A 34-year-old woman presented with a history of progressive left hypoglobus, enophthalmos, and diplopia in superior extreme gaze, compensated posturally. The patient did not report a history of trauma, sinonasal inflammatory disease, or any congenital deformity. Left eye enophthalmos was evident with no loss of visual acuity (Figure 1).

**Figure 2:**
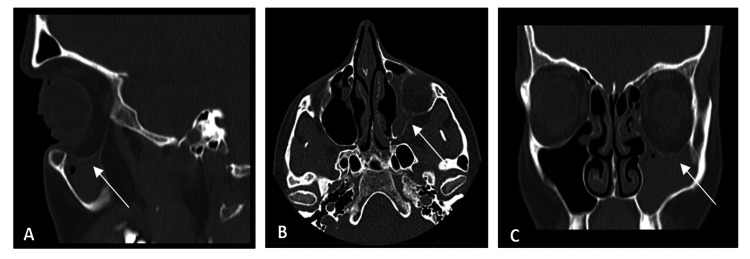
Preoperative CT scan showing the completely opacified left maxillary sinus, descent of the left orbital floor, and a decrease in the thickness of the inferior rectus muscle. A) Sagittal CT section showing the descent of the orbital floor and occupation of the left maxillary sinus (arrow). B) Axial section of the CT showing atelectasis of the left sinus volume (arrow) compared to the contralateral. C) Coronal section of the CT showing on the left side the smaller size of the inferior rectus muscle, the bone thinning of the orbital floor, and the occupation of the maxillary sinus (arrow). CT: Computed tomography

Computed tomography (CT) revealed left maxillary sinus opacification with secondary deformity of the orbital floor, medial orbital wall, and left inferior rectus muscle (Figure 2). An inferior bowing of the roof of the left maxillary sinus was seen on CT with an increased orbital volume.

**Figure 3:**
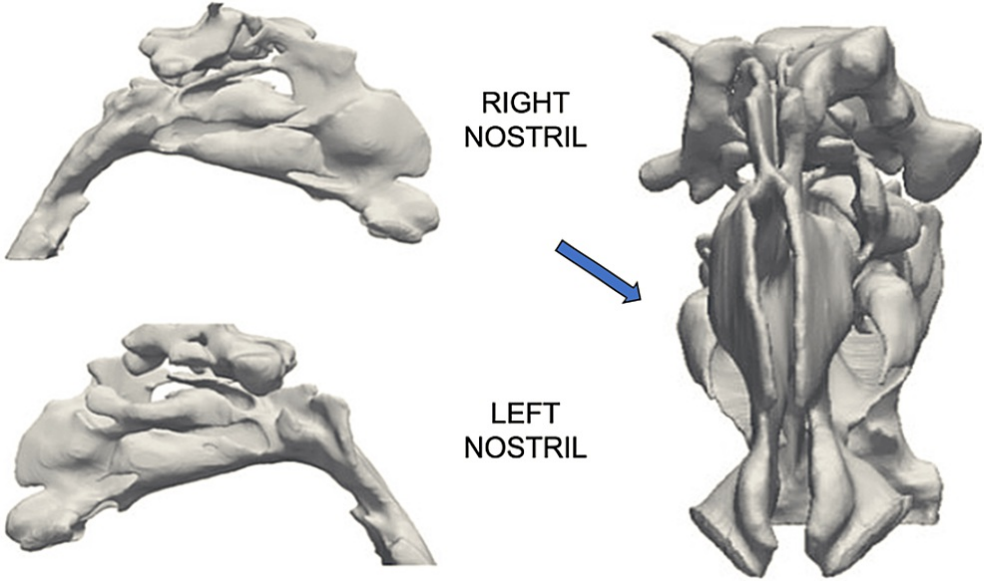
Nasal flow geometry. Right and left nostrils with symmetrical geometry and the absence of septal deviation. Sphenoid sinuses are represented, indicating permeability, but absence of representation of maxillary sinuses, indicating blockage (arrows).

Three-dimensional nasal airflow studies were conducted to target specific areas of hypoventilation, hypopressure, and atelectasis (Figure 3).

**Figure 4:**
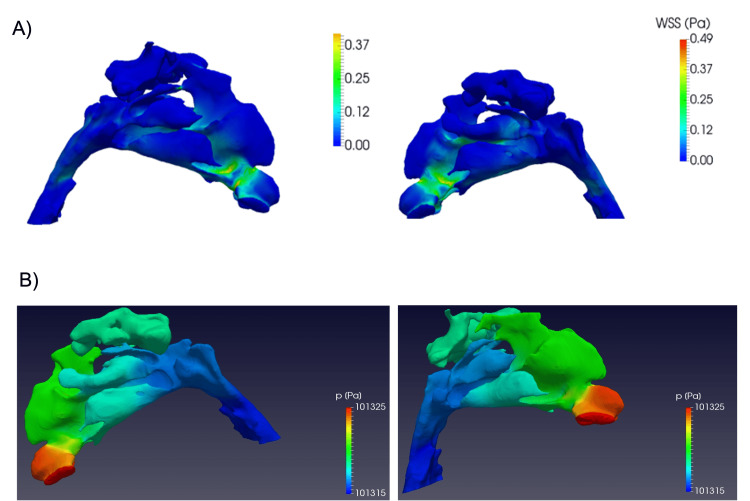
(A) WSS values that show nasal obstruction in the left nostril. (B) Flow pressure in both nostrils. WSS: Wall shear stress

High levels of wall shear stress (WSS) on the valve or outside the valve are indicative of nasal obstruction. The WSS in the post-valvular region of the left nostril is increased above normal reference values (>0.25 Pa), indicating nasal obstruction. In the nasal cavity of the right nostril, outside the valve, the values obtained are greater than 0.25, which is the threshold reference value to indicate nasal obstruction (Figure 4A). The difference in flow pressure in both nostrils stands out, which drops sharply in the anatomical region of the drainage ostia of the maxillary sinuses, with the decrease in pressure normally being more gradual throughout the nasal cavity (Figure 4B).

**Figure 5:**
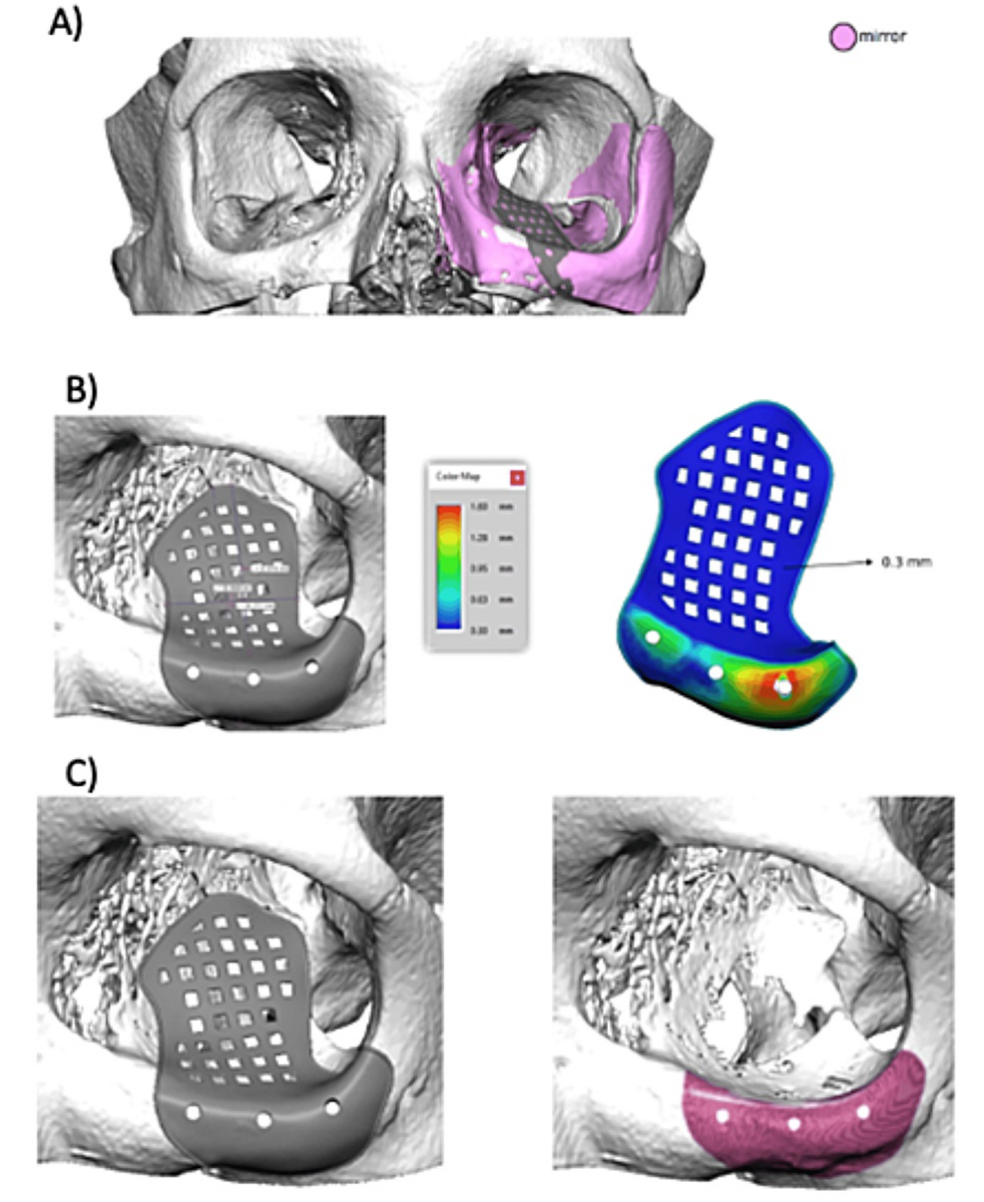
PSI design based on the mirrored image of the healthy orbit. A) Volumetric reconstruction of the pathological orbit with the healthy one. B) Design of the PSI and study of the bone contact areas (in red are the areas of greatest contact, coinciding with the screw fixation holes). C) Detail of the PSI design in the infraorbital rim. PSI: Patient-specific implant

The difference in flow pressure in both nostrils stands out, which drops sharply in the anatomical region of the drainage ostia of the maxillary sinuses, with the decrease in pressure normally being more gradual throughout the nasal cavity (Figure 5).

The overall flow rate is within normal values (7-12 L/min) in calm breathing. The nasal flow rate partitioning was 4.32 L/min in the left nostril (50.95%) and 4.16 L/min in the right nostril (49.05%). The flow rate is distributed in a very balanced manner and with normal values in each nostril independently (>3.5 L/min in both). Normal nasal resistance varies between 0.3 and 0.5 Pa s/cm^3^, with multiple variations existing between individuals. A resistance above 0.80 Pa s/cm^3^ is considered a diagnosis of nasal obstruction. In our study, a value of 0.71 Pa s/cm^3^ was obtained in the anterior rhinomanometry, which is not diagnostic of nasal obstruction, although the above values are considered normal.

**Figure 6:**
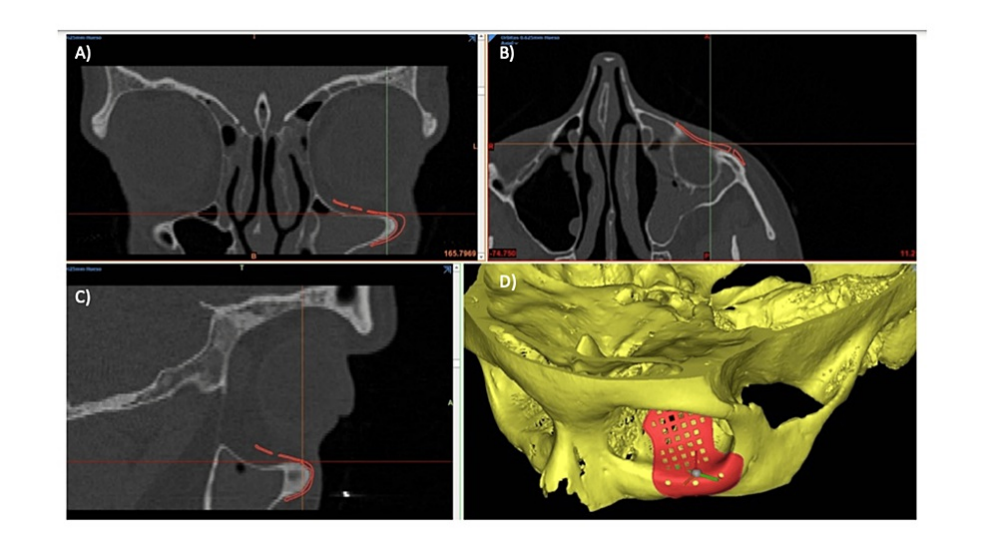
Adaptation of PSI in VSP. A) Coronal view; B) axial view; C) sagittal view; and D) tridimensional view. PSI: Patient-specific implant; VSP: Virtual surgical planning

After the nasal flowmetry study, three-dimensional (3D) printing models were made for the reconstruction of the affected orbit using the mirror image of the healthy contralateral orbit (Figure 5). Preoperative CT data were loaded into pre-surgical planning software (iPlan 3.0.5 BrainLab, Munich, Germany) (Figure 6). A virtual orbit was created based on the unaffected side, and a titanium patient-specific implant (PSI) was designed considering defect size and required plate thickness for orbital volume restoration. After final approval, the PSI was manufactured.

The patient’s surgery was planned collaboratively by both the otolaryngologist team and the oral and maxillofacial surgery (OMFS) team in a single procedure. Functional endoscopic sinus surgery (FESS)-assisted middle meatus antrostomy (MMA) followed by PSI reconstruction for volume and symmetry orbital restoration was planned, assisted by surgical navigation and intraoperative CT.

**Figure 7:**
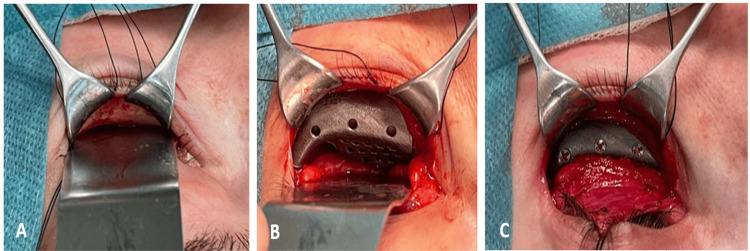
Intraoperative images of PSI placement. A) Exposure of the orbital rim and floor through a transconjunctival approach. B) Passive PSI adaptation without screw fixation. C) Osteosynthesis of the PSI with screws prior to closure. PSI: Patient-specific implant

First, a MMA and stripping of the nasal mucosa were performed using a FESS approach. Endoscopic vision confirmed left maxillary retraction, with inferior displacement of the orbital floor with obstruction of the left maxillary sinus meatus. Subsequently, volumetric reconstruction of the affected orbit was performed by placing the PSI through a transconjunctival approach without canthotomy (Figure 7).

Navigation ensured correct implant positioning and verified intraoperatively using a navigation probe and the intraoperative CT.

**Figure 8:**
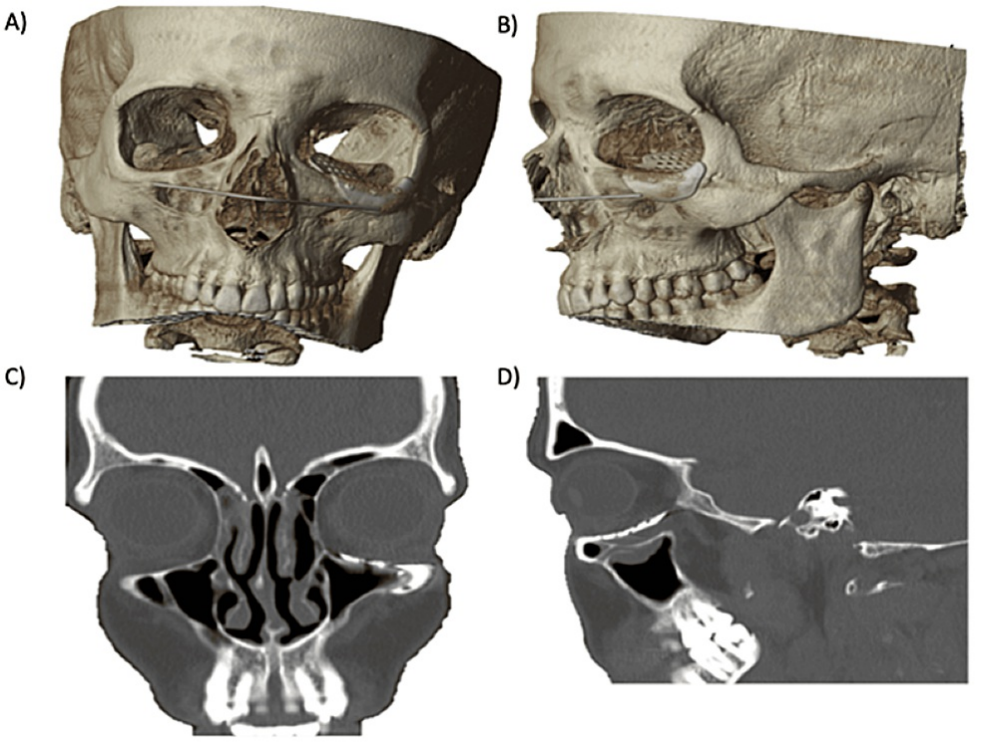
Postoperative CT scan, coronal and sagittal views with 3D reconstruction, showing PSI adaptation. A) 3D reconstruction, frontal view; B) 3D reconstruction, lateral view; C) correction of orbital floor asymmetry in CT coronal slice; and D) PSI adaptation in CT sagittal slice. CT: Computed tomography; 3D: Three-dimensional; PSI: Patient-specific implant

During follow-up, no late complications occurred, and results remained stable and aesthetically satisfactory one year after surgery. Subsequent postoperative CT scans showed no changes in the position of the left orbital floor reconstructive mesh (Figure 8).

Digital imaging and communication in medicine (DICOM) data imported into iPlan 3.0 (BrainLAB, Feldkirchen, Germany) confirmed implant orbital position using fusion tools, superimposing preoperative and postoperative CT scans with high accuracy.

**Figure 9:**
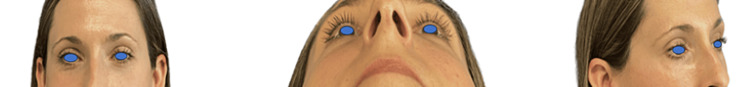
Postoperative result one year after surgery.

The patient demonstrated restored nasal airflow pressures, rectified inferior rectus muscle morphology, improved motility without experiencing diplopia, and a normalization of hypoglobus and enophthalmos (Figure 9).

**Figure 10:**
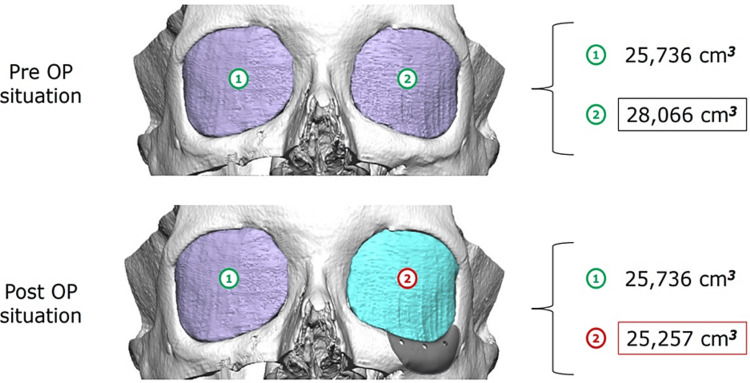
Preoperative and postoperative volumetry, showing the restoration of the volume in the affected orbit.

Significantly, orbital volume restoration was observed, with preoperative affected orbital volume at 28.066 cm^3^ (slightly increased compared to the healthy side's 25.736 cm^3^) and postoperative volume at 25.257 cm^3^, nearly matching the healthy side, with a total reduction of 2.809 cm^3^ (Figure 10).

## Discussion

Mucocele of the maxillary sinus causing enophthalmos was first described by Montgomery in 1964 [1] as CMA secondary to centripetal retraction of the sinus walls. Later, in 1994 Soparkar et al. [2] proposed the term SSS.

It is a rare entity that affects both sexes equally, generally between the third and fifth decades of life [3,4]. It consists of a spontaneous collapse of the walls of the maxillary sinus with the consequent descending of the floor of the ipsilateral orbit.

Clinically, it is characterized by progressive enophthalmos and hypoglobus with hypoplasia of the maxillary sinus and resorption of the orbital floor. Diplopia and orbital asymmetry may appear in the absence of other sinus symptoms. In fact, some authors [5,6] consider the presence of sinonasal symptoms an exclusion criterion. Exceptionally, a rare case has been described with an association of facial hypoesthesia [7] and even abnormal signs of the eyelids (retraction, ptosis, absent fold) and dry eye due to lagophthalmos [8]. Although it follows a progressive course, it can evolve rapidly as Jacobs et al. [9] reported on a patient with evidence of significant maxillary sinus remodeling over a period of 5.5 months.

Although it is generally diagnosed when diplopia appears secondary to the collapse of the orbital floor, cases without enophthalmos, with negative pressure in the maxillary sinus and collapse of the walls of the maxillary sinus visible on imaging tests, are considered an early stage. It usually affects unilaterally [3,4], without right or left predominance, although exceptionally some cases of bilateral involvement have been described [10,11].

Due to its rarity, its etiology, diagnosis, and treatment are uncertain. Patients usually deny pre-existing sinus disease or orbitofacial trauma, but cases associated with these have been described. Some authors consider it in the context of long-term asymptomatic maxillary sinusitis [6,12].

The pathophysiology remains uncertain. Initially, it was considered idiopathic. Some authors suggested that SSS occurs in patients with a congenital hypoplastic maxillary sinus when an infection is acquired [3,13]. It is currently widely accepted that it is due to an obstruction of the osteomeatal complex, which produces hypoventilation of the sinus and accumulation of secretions that creates a negative pressure that leads to atelectasis of the sinus with a downward displacement of the orbital floor [3,6,12,14].

The differential diagnosis should include facial hemiatrophy, progressive lipodystrophy, Horner syndrome [3,6], sinus hypoplasia, and CMA. Sinus hypoplasia, unlike SSS, is stable and congenital. SSS and CMA are terms that have been used interchangeably [15,16]. CMA consists of a persistent and progressive decrease in the volume of the maxillary sinus secondary to the inward curvature of the sinus walls. Some authors consider them to be the same entity, while others define them as two separate clinical entities, and SSS represents the last stage of CMA when deformities and visual alterations occur [6,17] or even a subtype of CMA [18]. To date, our study is the first that has carried out a nasal flowmetry study to dynamically diagnose the key areas of obstruction and hypoventilation to perform targeted treatment during FESS. SSS should be considered in the differential diagnosis of facial asymmetry [19].

Imaging techniques are essential, mainly CT and magnetic resonance imaging (MRI) [3,6]. Dynamic nasal flowmetry is not routinely used although it provides dynamic and functional information that cannot be obtained with CT or MRI. Radiologically, chronic sinusitis and maxillary sinus hypoplasia are the conditions that most frequently enter the differential diagnosis with SSS. The diagnosis is made after comprehensive imaging studies showing a reduction in the volume of the maxillary antrum and retraction of all or most of the maxillary walls [18-20]. The goal of treatment is to restore natural sinus ventilation and restore orbital volume. The traditional approach described by Soparkar et al. consisted of the Caldwell-Luc procedure [2,6], with the creation of a large antrostomy. Nowadays, maxillary endoscopic antrostomy and uncinectomy with FESS represent the gold standard of treatment to restore normal sinus ventilation [6], but there is no consensus on the management of enophthalmos. The main controversy in the literature remains the need to perform orbital reconstruction and, if so, the appropriate timing [6-12].

Some studies report that there is no need for orbital floor management after restoration of adequate maxillary sinus drainage. Sometimes, restoration of maxillary sinus ventilation is sufficient to improve enophthalmos and hypoglobus as it is believed to stop the bone resorption process. Rosso et al. [6] carried out a systematic review of the literature regarding its definition, diagnosis, and surgical approach. They conclude that isolated FESS seems to be the first treatment option since, in postoperative follow-up, orbital floor retraction tends to spontaneously reverse with clinically satisfactory results. Similarly, Sivasubramaniam et al. [17] performed a retrospective review of 23 cases of CMA managed with endoscopic uncinectomy and antrostomy alone where 22 of the 23 patients had partial resolution supporting the approach of delaying orbital floor reconstruction. Some authors are in favor of a two-stage approach, performing orbital reconstruction in a deferred manner. They highlight the advantages of this approach in avoiding the risk of implant infection and excessive overcorrection of the position of the eyeball. Babar-Craig et al. [18] recommend waiting at least six months after performing the antrostomy, since in their group of patients only two out of 16 required reconstructions of the orbit. Others believe that a single surgical approach is more convenient with the aim of rapid rehabilitation [4,6,19,20]. Although FESS can stop disease progression, it does not have to occur. In favor of this premise, Clarós et al. [20], in a retrospective study of 15 cases of patients undergoing combined treatment of endoscopic sinus surgery and reconstruction using Medpor® orbital floor implant, reported excellent results with no side effects observed. Furthermore, they report that, in their group of patients, the patient's main complaint was visible facial asymmetry, considering this a reason in favor of orbital reconstruction in a combined surgery. Soparkar [2] in a review of his 68 procedures over seven years insisted on a combined approach. Similarly, Arnon et al. [19] reported a case that presented facial asymmetry without diplopia that was treated surgically with a combined approach, performing FESS and orbitotomy with the insertion of an orbital implant. Sesena et al. [4] presented their experience with three cases treated with a single-stage procedure with excellent results reconstructed mainly with Medpor® implants, without infection or overcorrection in the 12 months after surgery. They suggest that a balance between the opposing pressures exerted by the soft tissues of the reconstructed orbit and the reexpanded sinus leads to a stable outcome.

In the literature, different materials have been described for the reconstruction of the orbital floor, from autologous grafts from iliac bone fragments to artificial implants made of biocompatible materials, including filling with autologous fat [3-14]. Preformed and non-preformed orbital implants, commonly used in the reconstruction of isolated orbital wall fractures, have also been applied for reconstruction in patients with SSS [11]. However, the three-dimensional involvement of the orbital volume accompanied by the bone remodeling that takes place in SSS makes it necessary to use more advanced techniques. Baig et al. [11] described for the first time the use of a PSI in this pathology. The use of personalized implants allows for obtaining an optimal aesthetic result and a reduction in surgical time, avoids complications at the graft donor site, and reduces morbidities associated with another general anesthesia procedure and hospital stay. Furthermore, Bonavolontà et al. [5] and Arnon et al. [19] reported the use of a virtual planning and navigation system to assist and guide surgery. All of this allows these results to be optimized, making them more predictable and visualizing at the time of surgery the correct placement of the mesh based on the previous planning carried out. In this way, we can reduce intraoperative errors and surgical time, facilitating the surgical procedure. Following this procedure, Raveggi et al. [12] presented two patients successfully treated with PSI and intraoperative navigation.

Consistent with previous reports [5,12,20], we believe that PSI, virtual surgical planning (VSP), surgical navigation, and intraoperative CT offer significant advantages compared to conventional surgical techniques. However, they are expensive techniques, and they are not available in many centers. In our opinion, the surgeon must individualize each case and discuss the expectations of the surgery with the patient.

## Conclusions

Optimizing both aesthetic and functional outcomes in complex volumetric defects, particularly those observed in SSS, can be achieved through orbital reconstruction utilizing PSI. The simultaneous management of maxillary sinus and orbital reconstruction within a single surgical session avoids the need for a second surgical procedure with a predictable outcome.

The integration of VSP, PSI, and intraoperative CT scan and navigation presents significant advantages over traditional surgical techniques. These advanced methods offer predictable results, heightened precision, and optimal volumetric reconstruction.
